# Validation of a patient‐specific quality assurance tool for offline analysis of treatment deliveries

**DOI:** 10.1002/mp.70161

**Published:** 2025-11-25

**Authors:** Marie Nasrallah, Michele Zeverino, Edward Chao, Dylan Casey, Fernanda Herrera, François Bochud, Raphaël Moeckli

**Affiliations:** ^1^ Institute of Radiation Physics Lausanne University Hospital Lausanne Vaud Switzerland; ^2^ Accuray Incorporated Madison Wisconsin USA; ^3^ Radiation Oncology Department Lausanne University Hospital Lausanne Lausanne Switzerland

**Keywords:** Adaptive radiation therapy (ART), Leaf open time (LOT), Patient specific quality assurance (PSQA), Tomotherapy

## Abstract

**Background:**

In radiation therapy, Patient‐Specific Quality Assurance (PSQA) is used to ensure safety and accuracy of treatment deliveries, which is paramount to control the disease while safeguarding healthy tissues. In common clinical practice, this involves comparing the planned dose to a dose measured in a phantom prior to the treatment. However, the emergence of Adaptive Radiation Therapy (ART) modalities challenges the PSQA workflow. This is especially the case in online ART workflow, where the patient is maintained immobilized on the treatment couch during treatment adaptation and delivery. Furthermore, for treatment plans using motion management, the plan is adapted in real time to encounter target motion. In these cases, the traditional phantom‐based pre‐treatment PSQA is impractical, and the development of substitute strategies is needed.

**Purpose:**

To validate a PSQA tool for the Radixact® units, which allows phantomless post‐delivery verifications.

**Methods:**

In tomotherapy, the dose is optimized by modulating the Leaf Open Times (LOTs) of the Multileaf Collimator (MLC). A metric based on the actual LOTs was developed to verify the accuracy of the dose delivered to the target and to the Organs At Risk (OARs). To validate the metric, treatment plans of prostate and Head and Neck (H&N) patients were modified by introducing errors in the LOT sinogram and subsequently delivered to a phantom. Based on these deliveries, LOT‐based metric indices were calculated and their sensitivity to clinically relevant errors were analyzed and compared to the standard benchmark gamma index metric. The clinically relevant errors included category 1 errors, when measured outputs don't remain within 3% of the established baseline, and category 2 errors, when the clinical goals are not fulfilled.

**Results:**

The LOT‐based metric outperforms the gamma index metric in predicting dose deviations and clinical goal deviations. It also demonstrates superior performance in classifying acceptable and unacceptable plans in terms of category 1 error detection for H&N patients, while its performance in category 1 error detection for prostate cases and category 2 error detection for both groups is comparable. The LOT‐based metric, with optimal cutoffs, outperforms the gamma index metric in terms of both sensitivity and specificity for category 1 and 2 error detection in prostate cases, while the gamma index metric outperforms the LOT‐based metric in term of sensitivity for category 2 error detection in H&N cases.

**Conclusions:**

This study establishes the LOT‐based metric as a reliable tool for pretreatment PSQA, serving as a substitute for the conventional gamma index metric, while additionally offering the advantage of fraction‐by‐fraction monitoring of treatment delivery quality.

## 1 INTRODUCTION

Ensuring a safe and accurate dose delivery stands as a priority in Radiation Therapy (RT). To guarantee a suitable agreement between the prescribed dose and the dose delivered by the Treatment Delivery System (TDS), national and international guidelines recommended performing Patient‐Specific Quality Assurance (PSQA).^1^, ^2^, ^3^, ^4^

To verify delivery accuracy, PSQA generally involves delivering the treatment plan on a phantom prior to the treatment. The dose predicted by the Treatment Planning System (TPS) is recalculated on a phantom geometry and the treatment plan is subsequently delivered to the phantom. The dose measured in the phantom is compared to the predicted dose with a metric, in most cases the gamma index metric.^5^ Despite its widespread use in clinics, this PSQA workflow has some limitations. Phantom‐based measurements are time‐ and effort‐intensive. The phantoms and detectors used for measurements show angular and energy dependencies, which limits the delivery verification.^6^, ^7^, ^8^, ^9^ In addition, pre‐treatment PSQA does not provide any insight into the errors that may occur during treatment delivery, such as patient anatomy changes, machine delivery errors, incorrect plan delivery or accidental plan modification.^10^, ^11^ Moreover, many studies observed that the gamma index metric may fail to identify clinically relevant errors.^12^, ^13^, ^14^, ^15^, ^16^, ^17^, ^18^, ^19^, ^20^, ^21^

Online Adaptive Radiation Therapy (ART) and real time motion tracking modalities, which allow for adaptation of the treatment plan according to the daily anatomical changes of the patient, introduce new challenges for PSQA. In online ART workflow, Organs At Risk (OARs) and target volume recontouring, along with plan re‐optimization and treatment delivery are conducted while the patient remains immobilized on the treatment couch.^22^, ^23^, ^24^ In real‐time motion tracking, the position of the target is continuously monitored and the beam is adjusted in real‐time to compensate for target motions. As the actual target position cannot be known prior to the treatment, the plan commanded by the TDS during delivery differs from the planned one. Therefore, verifying the commanded treatment plan for these modalities is not feasible with pre‐treatment phantom‐based measurements, which highlights the necessity to develop more appropriate PSQA methods. To overcome these complexities, some studies investigated the possibility to use predictive secondary dose calculations^25^ periodic dose measurements^26^ machine learning models^27^, ^28^ or statistical methods.^29^

Tomotherapy units (Accuray Inc, Sunnyvale, USA) are equipped with offline ART^30^ (PreciseART®) and motion management^31^ (Synchrony®) modalities. Additionally, an online ART solution is currently under development.^32^ In this context, Zhong et al.^33^ developed an independent dose calculation method to verify deformable dose accumulation for PreciseART® PSQA. Trujillo‐Bastidas et al.^34^ implemented a dynamic phantom‐based method for PSQA of Radixact Synchrony® treatments using film and array detectors. Tomotherapy utilizes a Multileaf Collimator (MLC) to modulate the intensity of a fan beam. The rotational delivery is divided into 51 segments per each gantry rotation, referred to as projections. During each projection time interval, the TPS determines the duration for which each of the 64 leaves remain open, referred to as the Leaf Open Time (LOT). The LOT values are influenced by the modulation factor, which reflects the complexity of the fluence pattern, and the pitch which reflects the longitudinal spacing between successive gantry rotations. A high modulation factor leads to greater maximum‐to‐mean LOT ratio, while a lower modulation factor decreases this ratio.^35^ Binny et al.^36^ showed that lowering modulation factor results in reducing the treatment plan mean LOT, while higher modulation factor is associated with increased treatment delivery time. A reduction of the pitch value increases the beam overlapping over the longitudinal slice, allowing for a fast gantry rotation and reducing the projection time^37^ while a higher pitch reduces overlap and leads to higher projection time. Some studies demonstrated that discrepancies between planned and actual MLC LOTs can result in clinically significant dosimetry inaccuracies in helical tomotherapy.^38^, ^39^ Sevillano et al.^40^ developed an independent in‐air LOT calculation method based on the MegaVoltage (MV) detector mounted on the gantry ring.^40^, ^41^, ^42^, ^43^ Thiyagarajan et al.^44^ used 3D dose reconstruction based on LOTs measured with the MV detector signal to verify delivery accuracy in‐air for PSQA purposes and showed that this method is particularly valuable for total marrow irradiation, for which there is a lack of suitable measurement devices. Han et al.^43^ also developed a phantomless PSQA tool based on LOT calculations. In their approach, they calculated the 3D delivered dose within the patient's anatomical geometry, based on LOTs calculated with MV detector data, and they then compared the calculated dose to the planned one with the gamma index metric. Chen et al.^42^ and Schopfer et al.^41^ extended the MV detector LOT measurements method to in vivo MLC performance verifications. Resuming the Schopfer et al. method^41^ the detector signal is processed and normalized to the maximum signal value near the projection center for each leaf within each projection. The actual LOTs are then calculated by measuring the width of the corresponding signal segments. To calibrate and validate this method against the ground truth, in phantom ionization chamber measurements were performed.^45^ Pisaturo et al.^46^ developed a transit dosimetry verification method based on the MV detector signal acquired during treatment delivery. Schopfer et al.^47^ developed a LOT‐based metric for PSQA and demonstrated that the mean discrepancy between the planned and the measured LOTs correlates with the dose deviation in the Planned Target Volume (PTV). However, the correlation between this metric and the dose deviations in the OARs was not demonstrated.

This work aims to enhance the LOT‐based metric, based on Schopfer et al. works^41^, ^47^ by developing new indicators for the target and OARs and by analyzing their ability to detect clinically relevant errors. For this, a script was implemented to pinpoint the leaves responsible for primary dose deposition in the OARs and the OARs indicators were defined as the mean discrepancy between planned and actual LOTs of the pinpointed leaves. The enhanced LOT‐based metric was applied to clinical scenarios for prostate and Head and Neck (H&N).

The LOT‐based metric allows delivery accuracy verifications in‐air prior to the treatment, positioning it as an alternative to the conventional clinical PSQA approach. Moreover, the MV detector data acquisition can be performed in‐real time, with the patient on the treatment couch and for each fraction. In this context, data processing allows for post‐treatment verification of the delivery, without incurring additional dose to the patient. This method permits phantomless pre‐treatment verifications for conventional and offline ART workflows and post‐treatment delivery verifications for conventional, offline and online ART as well as motion management workflows. While it does not support pre‐treatment verification of the adapted plan in online ART and motion management, this technique is designed for retrospective validation of the delivery, which may facilitate decision‐making regarding necessary inter‐fraction modifications.

## 2 MATERIALS AND METHODS

### 2.1 Treatment plans

Four prostate patients, each prescribed 39 fractions of 2 Gy per fraction to the PTV median volume, were randomly selected for this study. Similarly, four H&N patients, each prescribed 33 fractions of 2.12 Gy per fraction to the high‐risk PTV and 1.58 Gy per fraction to the low‐risk PTV were selected. For H&N cases, prescribed dose was normalized to the high risk PTV median volume. To ensure a dosimetric similarity, the H&N patients met the following criteria: oropharynx cases with bilateral irradiation, without metastasis in the lymph nodes, with simultaneous integrated boost, with similar distance between the PTV and the parotids and between the PTV and the brachial plexus. No specific criteria were used for the prostate patient selection. The parameters of the patient treatment plans are summarized in Table 1. In this study, all plans used 2.5 cm jaw opening. The pitch provides insight into the beam overlap among successive rotations and is calculated by dividing the couch translation by the field width. The modulation factor gives information on the MLC LOT modulation complexity and is defined as the longest leaf opening time in a plan divided by the average opening time of all non‐zero LOTs.^48^

**TABLE 1:** Patients treatment plans used in this study.

Patient	Treated site	Number of projections	Time per projection (ms)	Modulation factor	Pitch
P1	Prostate	444	347	1.764	0.430
P2	Prostate	771	347	2.202	0.287
P3	Prostate	394	347	1.855	0.430
P4	Prostate	739	300	1.921	0.287
P5	H&N	1625	347	2.709	0.287
P6	H&N	1338	347	3.214	0.287
P7	H&N	1311	347	2.687	0.287
P8	H&N	1263	347	2.554	0.287

### 2.2 Leaf Open Time (LOT) based metric

The primary function of the beamlets is to deliver dose to the PTV. Therefore, in this work, the mean discrepancy between the planned and the measured non‐zero LOTs was calculated and its sensitivity to clinically relevant errors occurring in the PTV for prostate cases and in the high‐risk PTV for H&N cases was evaluated. Hereinafter, the mean discrepancy of all non‐zero LOTs is denoted $\bar{\Delta LOT_{PTV}}\;$ for prostate cases and $\bar{\Delta LOT_{PTV\;high\;risk}}$ for H&N cases.

During treatment delivery, dose deposition also occurs to other structures, such as OARs. Consequently, while considering the development of a metric based on LOTs which is sensitive to dose deviations, it is of interest to identify the beamlets responsible for primary dose deposition in the Region of Interest (ROI). The approach is to pinpoint the leaves within the sinogram that deliver primary dose to the ROI. It is worth noting that this represents a first‐order approximation, focusing exclusively on the beamlets delivering primary dose to the OAR and omitting the beamlets responsible solely of scattering events.

A MATLAB® R2021a (MathWorks, Inc) script was implemented to ray‐trace the intersection between the beamlets and the OARs. For this purpose, the corresponding Digital Imaging and Communications in Medicine (DICOM) structure file and the raw data file containing the gantry and jaw positions were collected. Using the DICOM structure file, the script performed a projection of the OAR on the plane parallel to the MLC and passing through the isocenter, called the MLC plane. The MLC plane origin was centered on the isocenter, between the 2 central MLC leaves. The projected volume was then superposed with the LOT sinogram to determine the intersecting beamlets, taking into account the jaw and gantry positions during delivery (Figure 1). For each ROI, the mean LOT discrepancy between the planned and the measured LOTs was calculated for the intersecting beamlets and represented by $\bar{\Delta LOT_{\mathrm{OAR}}}$. Subsequently, its sensitivity to clinically relevant errors was evaluated.

**FIGURE 1:**
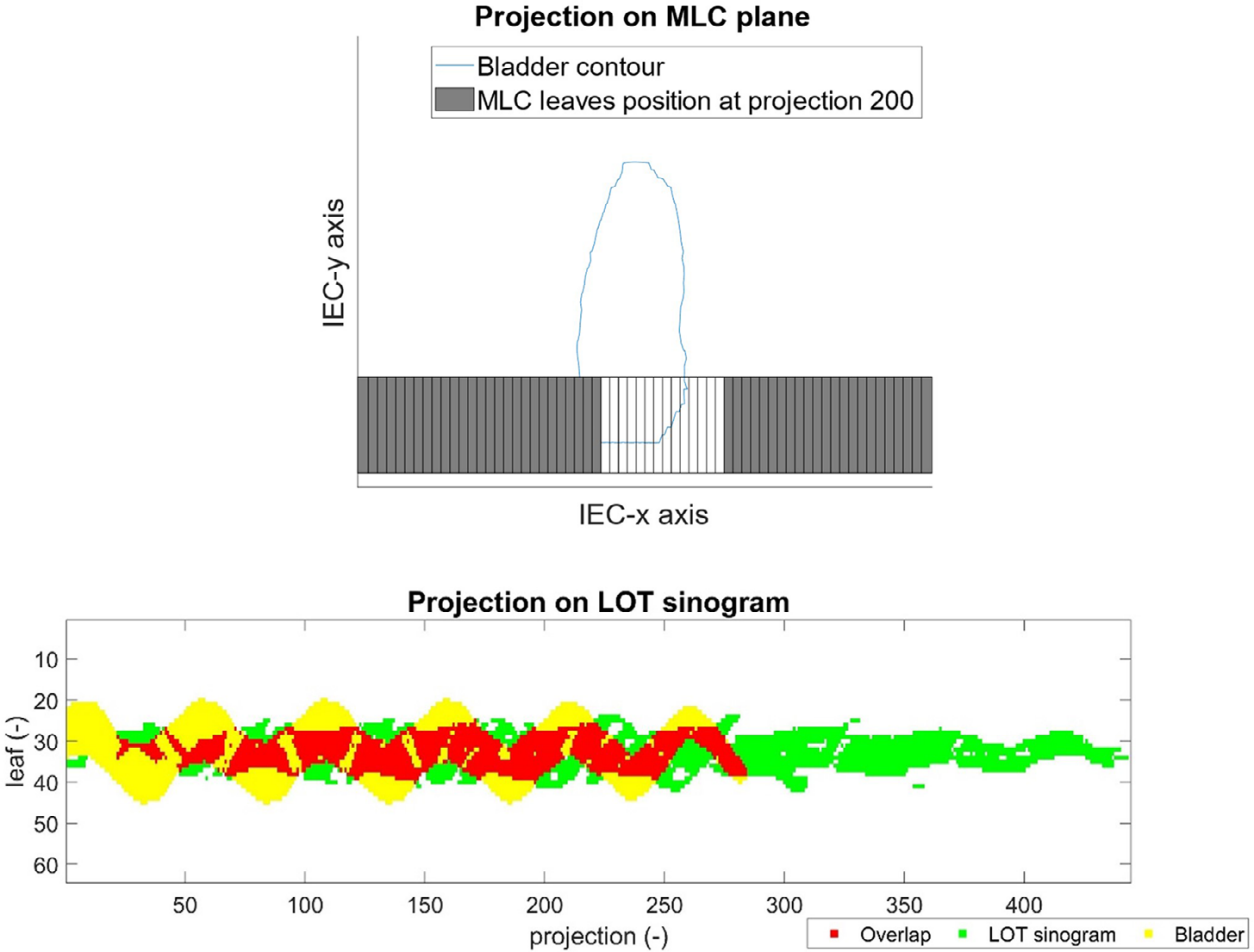
Illustration of the ray‐tracing method. The upper graph represents the projection of the bladder on the MLC plane at projection Nr 200; the gray filled rectangles represent the closed leaves and the empty rectangles represent the open leaves. The lower graph illustrates the overlap between the bladder and the Leaf Open Times (LOT) sinogram.

The same script was also used to calculate the mean LOT discrepancy between the planned and the measured LOTs of the intersecting beamlets for the Clinical Target Volume (CTV) for prostate cases, written $\bar{\Delta LOT_{\mathrm{CTV}}}$, for the high‐risk CTV for H&N cases, written $\bar{\Delta LOT_{\mathrm{CTV}\;\mathrm{high}\;\mathrm{risk}}}$, and for low‐risk PTV and CTV for H&N cases, written $\bar{\Delta LOT_{\mathrm{PTV}\;\mathrm{low}\;\mathrm{risk}}}$ and $\bar{\Delta LOT_{\mathrm{CTV}\;\mathrm{low}\;\mathrm{risk}}}$.

In this study, a global index was defined to account for all LOT‐based metric indices within a single value. This LOT‐based metric global index $\bar{\Delta LOT_{\mathrm{global}}}$ was defined as the maximum index value over all evaluated ROIs:

(1) $$\bar{\Delta \mathrm{LO}T_{\mathrm{global}}}=\max\left(\left\{\left|\bar{\Delta \mathrm{LO}T_{\mathrm{RO}I_{i}}}\right|,i=1,\cdots ,n\right\}\right)$$

where $\bar{\Delta LOT_{ROI_{i}}}$ is the index of the $i^{th}$ evaluated ROI.

### 2.3 Modified plans

To evaluate the ability of the LOT‐based metric indices to detect clinically relevant errors, three different types of delivery inaccuracies were artificially introduced in the LOT sinograms for the 8 selected treatment plans (Table 1): systematic errors in the entire sinogram, random error following Gaussian distribution in the entire sinogram, and systematic errors of the two central MLC leaves. These types of discrepancies were applied solely to non‐zero LOTs.

As an MLC interlock system (see more details in Supporting Information) stops irradiation if one LOT error exceeds 70 ms, systematic LOT discrepancies of −70 ms, −10 ms, −2 ms, +2 ms, +10 ms, and +70 ms were introduced across the entire LOT sinogram. Overall systematic errors between −70 ms and +70 ms are the worst‐case scenarios, which would bypass the interlock system detection.

According to the central limit theorem and given the large number of LOTs delivered (between 3'209 and 5'898 for prostate cases and between 8'742 and 14'838 for H&N cases) for the selected reference treatment plans (Table 1), the sample mean of LOT discrepancies tends toward a Gaussian distribution. Therefore, to test potential LOT discrepancies scenarios, random errors were generated based on this distribution. To determine the Gaussian distribution LOT standard deviations, the reference treatment plans were delivered twice on an Octavius® II phantom (PTW, Freiburg, Germany). The corresponding MV detector signal was collected after each in‐phantom delivery and used to measure the actual LOTs. Although no standard deviations greater than 5.8 ms were observed^40^ we generated plans with LOT discrepancies following a Gaussian distribution centered at 0 ms, with standard deviations between 1.0 ms and 35.0 ms, to account for potential cases where LOT values approach the MLC interlock threshold (70 ms).

The most frequent MLC failure according to the manufacturer consists of one or two leaves remaining open systematically longer or shorter than expected during the entire delivery. For symmetric targets extents in the left‐right direction as for the prostate and bilateral H&N cases, the two central leaves are responsible for most of dose deposition into the patient. Therefore, systematic errors in the two central leaves of −70 ms, −10 ms, −2 ms, +2 ms, +10 ms, and +70 ms were introduced. Systematic errors of −70 ms and +70 ms in the two central leaves are the worst‐case scenarios for this type of error, which would bypass the interlock system detection.

After introducing errors, corrections were applied to ensure that no LOT exceeded the duration of one projection or went below 0 ms. Specifically, if a LOT exceeded the projection time, it was adjusted to match the projection time. Similarly, if a LOT felt below 0 ms, it was set to 0 ms. These corrections guaranteed that all LOTs remained within the valid range between 0 ms and the projection time.

In the following sections, the clinical treatment plans are referred to as “reference” plans and the corresponding LOT sinograms and LOTs are denoted $Sino^{\mathrm{ref}}$ and $LOTs^{\mathrm{ref}}$. The clinical treatment plans with introduced LOT discrepancies are referred to as “modified” plans and the corresponding LOT sinograms and LOTs are denoted $\mathrm{Sin}o^{\mathrm{mod}}$ and $LOTs^{\mathrm{mod}}$. The LOTs measured with the MV detector signal during deliveries are denoted $LOTs^{\mathrm{actual}}$, the corresponding sinogram $Sino^{\mathrm{actual}}$, and the corresponding plan the “actual” plan. If a specific ROI has been ray‐traced, namely OARs, CTV, PTV low risk, CTV high risk or CTV low risk, this is specified in the notation subscript. The part of a sinogram responsible for primary dose deposition in a specific ROI is denoted $Sino_{\mathrm{ROI}}$ and the corresponding LOTs are denoted $LOTs_{\mathrm{ROI}}$.

### 2.4 Clinically relevant errors

In this work, the ability of the LOT‐based metric indices to detect two different categories of clinically relevant errors were analyzed and compared to the Benchmark gamma index metric. To ensure accurate delivery, AAPM task group 198 recommends that measured outputs should remain within 3% of the established baseline^49^ In this work, the outputs of the delivered plans were assessed to determine if they fell within the ± 3% limit for various dose parameters listed in Table 2. Deliveries, for which all evaluated dose parameters were within ±3% of planned values were classified as “acceptable”, while those with at least one parameter outside this range were deemed “unacceptable”. A surpassing of this limit is referred to as category 1 error or “Relative dose delivery” errors. The category 2 error, referred to as the “Clinical goals achievement” error, classified deliveries that successfully met all evaluated clinical goals (Table 2) as “acceptable”, while those that failed to meet at least one clinical goal were classified as “unacceptable”. The different ROIs and their corresponding LOT‐based metric indices, the evaluated dose parameters for category 1 errors and the evaluated clinical goals for category 2 errors are summarized in Table 2.

**TABLE 2:** LOT‐based metric indices and corresponding dose parameters and clinical goals evaluated in this study. Dmax is defined as the dose within $0.03cm^{3}$ of the volume.

Treatment region	Target/ROI	LOT‐based metric index	Category 1 error: Evaluated dose parameter	Category 2 error: Evaluated clinical goal
Prostate	PTV; CTV	$\bar{\Delta LOT_{\mathrm{PTV}}};\bar{\Delta LOT_{\mathrm{CTV}}}$	D95	At least 95% of the volume at 95% of the prescription
			D05	At most 5% of the volume at 105% of the prescription
	Bladder	$\bar{\Delta LOT_{\mathrm{Bladder}}}$	D50	At most 50% of the volume at 65 Gy
			D25	At most 25% of the volume at 70 Gy
	Rectum	$\bar{\Delta LOT_{\mathrm{Rectum}}}$	D50	At most 50% of the volume at 50 Gy
			D20	At most 20% of the volume at 70 Gy
			D10	At most 10% of the volume at 75 Gy
H&N	PTV high risk; CTV high risk	$\bar{\Delta LOT_{\mathrm{PTV}\;\mathrm{high}\;\mathrm{risk}}}$ $\bar{\Delta LOT_{\mathrm{CTV}\;\mathrm{high}\;\mathrm{risk}}}$	D95	At least 95% of the volume at 95% of the prescription
			D05	At most 5% of the volume at 105% of the prescription
	PTV low risk; CTV low risk	$\bar{\Delta LOT_{\mathrm{PTV}\;\mathrm{low}\;\mathrm{risk}}}$ $\bar{\Delta LOT_{\mathrm{CTV}\;\mathrm{low}\;\mathrm{risk}}}$	D95	At least 95% of the volume at 95% of the prescription
	Parotid left; Parotid right	$\bar{\Delta LOT_{\mathrm{Parotid}\;\mathrm{left}}}$ $\bar{\Delta LOT_{\mathrm{Parotid}\;\mathrm{right}}}$	D50	At most 50% of the volume at 20 Gy
	Spinal canal	$\bar{\Delta LOT_{\mathrm{Spinal}\;\mathrm{canal}}}$	*D*_max_	At most $0.03\;cm^{3}$ at 40 Gy
	Brainstem	$\bar{\Delta LOT_{Brainstem}}$	*D*_max_	At most $0.03\;cm^{3}$ at 54 Gy
	Chiasm	$\bar{\Delta LOT_{\mathrm{Chiasm}}}$	*D*_max_	At most $0.03\;cm^{3}$ at 54 Gy
	Brachial plexus left; Brachial Plexus right	$\bar{\Delta LOT_{\mathrm{Brachial}\;\mathrm{plexus}\;\mathrm{left}}}$ $\bar{\Delta LOT_{\mathrm{Brachial}\;\mathrm{plexus}\;\mathrm{right}}}$	*D*_max_	At most $0.03\;cm^{3}$ at 55 Gy

### 2.5 Measurements

The reference treatment plans were delivered twice and each modified plan was delivered once with a Radixact® unit (see more details in Supporting Information) on the Octavius® II phantom, within which a PTW 2D‐array seven29 (PTV, Freiburg, Germany) was placed. After each in phantom delivery, the 2D‐array measurements and the raw data file containing the MV detector signal were collected simultaneously. For the 2D‐array measurements, a global correction factor was applied to compensate for the detector's angular response and point doses were recorded within the irradiated region. Acquiring both in phantom measurements and MV detector data from the same delivery permitted the calculation of the gamma passing rate from the 2D‐array data (by comparing the delivered to the reference dose) and the LOT‐based metric indices from the MV detector data (by comparing the actual to the reference LOTs) simultaneously. The 2D‐array measurement was imported in VeriSoft® software (PTW, Freiburg, Germany) to calculate the gamma passing rate.^5^ The passing rate threshold used in our institution for gamma metric analysis involves ≥ 95% of agreement with 3% /3 mm tolerance criteria (dose normalized to maximum value). The MV detector signal was processed to calculate $Sino^{\mathrm{actual}}$ and the corresponding LOT‐based metric indices, as summarized in Table 2. This approach allows for the comparison of the two PSQA metrics for the same delivery, which involves attenuating object in the beam path.

To track clinically relevant delivery errors occurring during delivery, an Accuray Inc. in‐house dose calculator was used to calculate the actual dose, based on the previously calculated $Sino^{\mathrm{actual}}$, that the patient would have received with the delivered plan. The jaws and gantry positions during delivery were also taken into account for the dose calculation. Additionally, the reference dose, initially optimized with the RayStation® (RaySearch Laboratories, Stockholm, Sweden) TPS was calculated with the Accuray Inc. in‐house dose calculator, using $Sino^{\mathrm{ref}}$ as well as the planned jaws and gantry positions. This was to ensure that the same dose calculation algorithm was applied as for the actual dose and to permit comparison between planned and delivered doses. The patient planning Computed Tomography (CT) images were used for dose calculations. The doses were then imported into RayStation® for DVH parameters calculation and visualization. A verification was conducted to ensure the DVHs superposition of both the planned dose and the reference dose calculated with the Accuray Inc. in‐house dose calculator.

To analyze the delivery consistency of the reference plans, $Sino^{\mathrm{actual}}$ were compared to the corresponding $Sino^{\mathrm{ref}}$ and unpaired two‐sample t‐tests were performed. To ensure proper delivery of the modified plans, $Sino^{\mathrm{actual}}$ was compared to the corresponding $Sino^{\mathrm{mod}}$ (Figure S1 in Supporting Information).

For each delivery, the ability of the gamma passing rate and the LOT‐based metric global index (Equation (1)) to detect clinically relevant errors of categories 1 and 2 was analyzed. The categories 1 and 2 errors occurring during delivery were observed in RayStation® visualization, by extracting the evaluated dose parameters and clinical goals (Table 2) and by comparing these values to the reference values (the unmodified reference treatment plan).

The category 1 error detection was evaluated by calculating the relative discrepancy between the planned dose (the unmodified patient treatment plans) and the delivered dose for the evaluated dose parameters summarized in Table 2. Analysis of linear correlation between the LOT‐based metric indices and corresponding evaluated relative dose discrepancy parameters, as well as between the gamma passing rate and evaluated relative dose discrepancy parameters were performed by calculating the Pearson coefficients r and corresponding *p*‐values. For the analysis, only the data points with a dose discrepancy in the range of [−6%, +6%] were accounted for to ensure a good fit close to the ±3% dose threshold. We selected values near the 0% mean dose discrepancy, as small discrepancies are more likely to occur than larger ones. The ± 6% range was chosen to maintain symmetry around the ± 3% threshold, where plans with discrepancies between 0% and ± 3% are considered acceptable and those between ± 3% and ± 6% are considered unacceptable. Category 2 error detection was evaluated by calculating the clinical goals summarized in Table 2. Analysis of monotonic correlation between the LOT‐based metric indices and corresponding clinical goals, as well as between the gamma index metric and the clinical goals were performed by calculating the Spearman Rank's coefficients ρ and corresponding p‐values. To analyze the performance of the LOT‐based metric and the gamma index metric in detecting the deliverability of plans in terms of category 1 and category 2 errors, Receiver Operating Characteristic (ROC) curves were plotted and Delong tests^50^ were conducted to compare the Area Under the Curves (AUCs) of the two metrics. Youden's index^51^ was used to determine the optimal cutoffs, which maximize the difference between the true positive rate and the false positive rate for optimal classification of acceptable and unacceptable cases. Additionally, the bootstrap method^52^ was applied by generating multiple resampled datasets, estimating the Confidence Intervals (CIs) for the ROC curves at the selected cutoffs, and assessing the difference in sensitivity and specificity between the two methods for the optimal as well as for the clinically used cutoffs.

## 3 RESULTS

### 3.1 Category 1 error: Relative dose delivery

All evaluated dose parameters of reference plan deliveries (with no introduced errors) listed in Table 2 successfully meet the ±3% dose limit, as recommended by the AAPM task group 198.^49^ Including the reference and the modified plan deliveries, 59.4% of the plans in the prostate cohort successfully meet the ±3% dose limit, while 40.6% have at least one dose parameter which does not meet this limit. For H&N cases, 51.6% of the plans fulfill the ±3% dose limit, while 48.4% have at least one dose parameter which does not meet this limit.

The first delivery of the patient P2 reference plan shows a gamma passing rate of 95.6%, successfully meeting the PSQA test requirement of a gamma passing rate above 95%, while the second delivery shows a gamma passing rate of 94% which misses the test requirement. Despite this, the plan passed the PSQA for the real treatment of the patient. For the other patients, all reference plan deliveries meet the test requirement, with gamma passing rates above or equal to 98%.

Figure 2 illustrates the relationship between the dose parameters and gamma passing rates (first column) and between the dose parameters and the LOT‐based metric indices (second column) for PTV D95 and rectum D10 of the prostate cases, while Figures S2 and S3 in Supporting information show these relationships for all evaluated dose parameters cited in Table 2 for each patient (P1 to P8). The red points represent the delivery of reference plans and the black points the delivery of modified plans. The points in the ±3% dose range fulfill the ±3% dose limit for the evaluated dose parameter while the points outside this range fail to achieve the limit. The plain black lines correspond to the linear fit, for which only the data points with a dose discrepancy in the range of [−6%, +6%] were accounted for to ensure a good fit close to ±3% dose threshold. The dashed red lines correspond to the 95% gamma metric cutoff used clinically.

**FIGURE 2:**
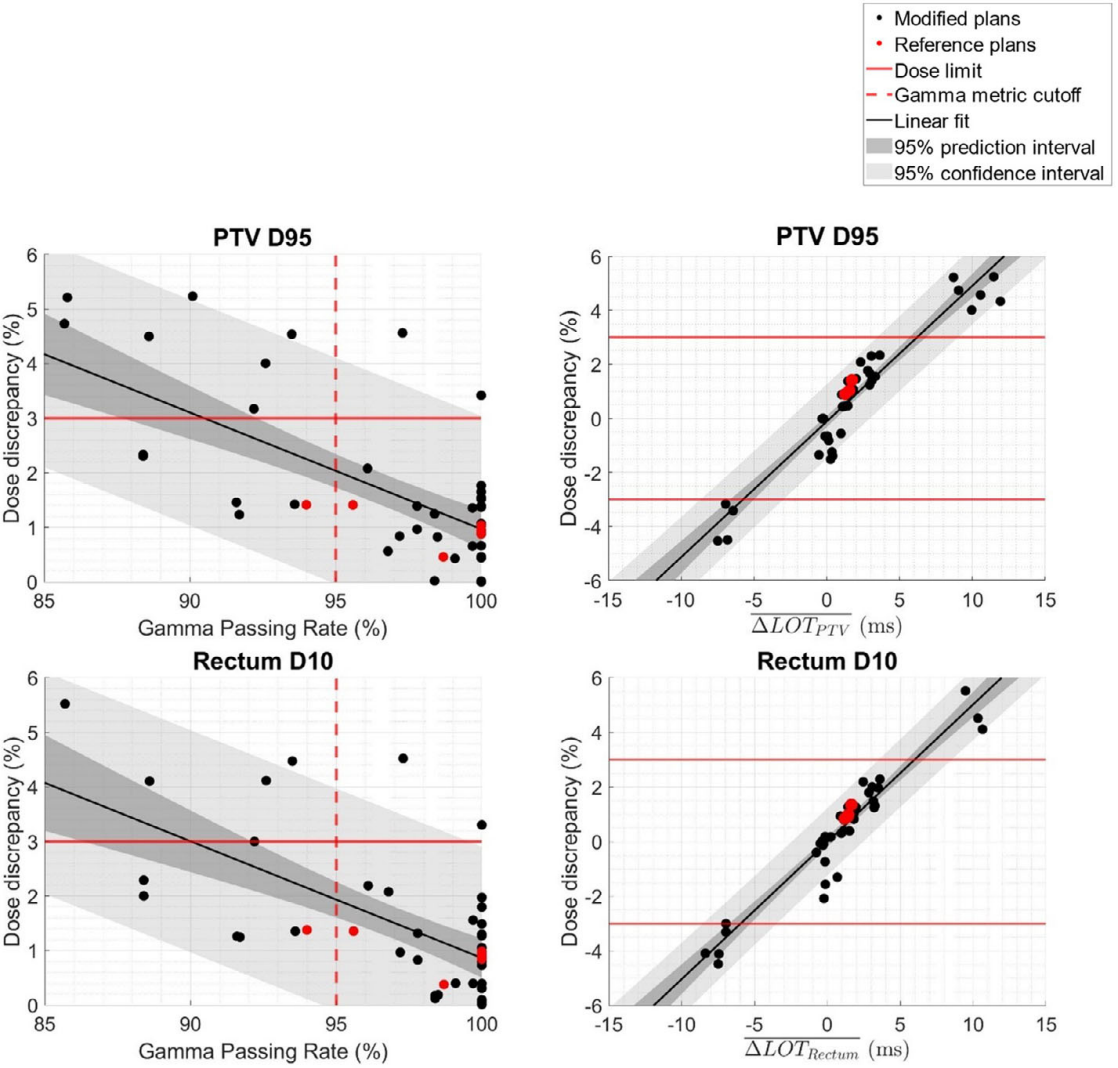
First column: Relationship between evaluated dose parameters and gamma passing rates for the PTV D95 (first line) and rectum D10 (second line) of prostate cases. Second column: Relationship between dose parameters and LOT‐based metric indices for the PTV D95 (first line) and rectum D10 (second line).

Pearson coefficients and corresponding *p*‐values show significant inverse linear correlation between dose parameters and gamma passing rates at 5% significance level (r∈[−0.97;−0.87], $p\in [5*10^{-42};5*10^{-21}]$ for prostate cases and r∈[−0.95;−0.86], $p\in [8*10^{-32};2*10^{-19}]$ for H&N cases). The results show significant linear correlations between dose parameters and LOT‐based metric indices at 5% significance level (r∈[0.94;0.98], $p\in [3*10^{-38};1*10^{-23}]$ for prostate cases and r∈[0.74;0.96], $p\in [6*10^{-28};2*10^{-08}]$ for H&N cases), except for the chiasm *D*_max_ for which the Pearson coefficient is undefined. This is because there are no intersecting leaves between the MLC and the chiasm, thus $\bar{\Delta LOT_{\mathrm{Chiasm}}}=0$ for each delivery, which leads to a division by 0 when calculating the Pearson coefficient.

### 3.2 Category 2 error: Clinical goals achievement

All reference plan deliveries successfully meet the evaluated clinical goals defined in Table 2. Including the reference and the modified plan deliveries, 59.4% of the plans in the prostate cohort successfully meet the clinical goals and 40.6% have at least one clinical goal non‐achievement. For H&N cases, 37.5% of the plans fulfill the clinical goals and 62.5% have at least one clinical goal non‐achievement.

Figure 3 illustrates the relationship between the gamma passing rate and two different clinical goals (first column), as well as between the LOT‐based metric indices and two different clinical goals (second column). Figure S4 and S5 in Supporting information illustrate this relationship for each evaluated clinical goals cited in Table 2 for each patient (P1 to P8). The dose constraints have been divided by the total number of fractions to display the constraint for a unique delivery. The red plain lines represent the acceptance levels. For the graphs related to the PTV, the points above this level fulfill the evaluated clinical goal while, for the graphs related to the rectum, the points below this level fulfill the evaluated clinical goal. The red dashed line represents the clinically used 95% passing rate cutoff. Spearman Rank correlation coefficients and corresponding p‐values show no significant monotonic correlation between clinical goals and gamma passing rates at a 5% significance level, except for the clinical goal “At most 5% of the volume at 105% of the prescription” for the PTV (ρ=−0.28, p=0.02) and the CTV (ρ=−0.26, p=0.04) in prostate cases, as well as for the clinical goal related to the brachial plexus left (ρ=−0.32, p=0.01) and for the clinical goal “At least 95% of the CTV high risk volume at 95% of the prescription” (ρ=0.41, p=0.001) in H&N cases. The correlation is undefined for the brainstem and the chiasm as 0% of the volume reaches the dose limits across all plans, leading to a division by zero when calculating the Spearman Rank correlation coefficient. The results show significant monotonic correlation between the clinical goals and the LOT‐based metric indices at a 5% significance level (ρ∈[0.52;0.93], $p\in [3*10^{-28};1*10^{-5}]$ for prostate cases and $\rho \;\in \;\left[0.41;0.93\right],p\in \left[5*10^{-28};7*10^{-4}\right]$ for H&N cases). The exception to this is the clinical goal related to the spinal canal (ρ=0.21, p=0.10) as 0% of the volume reaches the dose limit in all but one plan. The Spearman Rank correlation coefficient is undefined for the clinical goal related to the brainstem for both the gamma passing rates and the LOT‐based metric indices as 0% of the volume reaches the dose limit across all plans, and for the chiasm, which has no intersecting leaves with the MLC and 0% of the volume reaching the dose limit. These latter cases lead to a division by 0 when calculating the Spearman Rank correlation coefficient.

**FIGURE 3:**
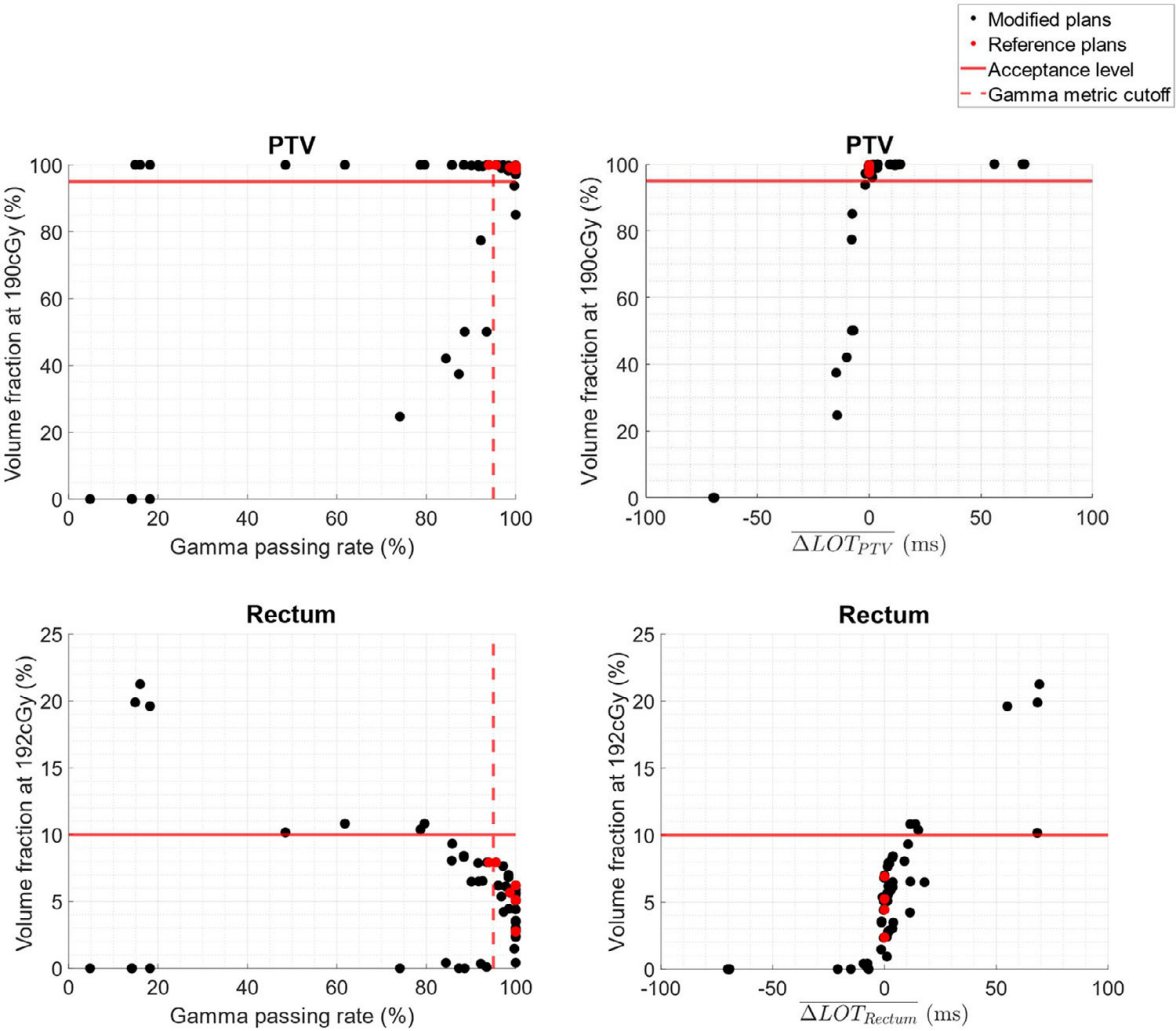
First column: relationship between the gamma passing rates and clinical goals. second column: relationship between the LOT‐based metric indices and clinical goals. The evaluated clinical goals are “At least 95% of the PTV volume at 95% of the prescription” and “At most 10% of the rectum volume at 75 Gy”.

### 3.3 ROC curves

Figure 4 displays the ROC curves for the LOT‐based metric global index $\bar{\Delta LOT_{\mathrm{global}}}$ (Equation (1)) and the gamma passing rates for category 1 and category 2 error detection. Sensitivity, specificity and corresponding 95% CIs estimated with the resampling bootstrap method are plotted at the bottom of Figure 4 and listed in Table S1 in Supporting information. Table 3 shows the AUCs, the p‐values related to the Delong test, and the optimal Youden cutoff.^51^

**FIGURE 4:**
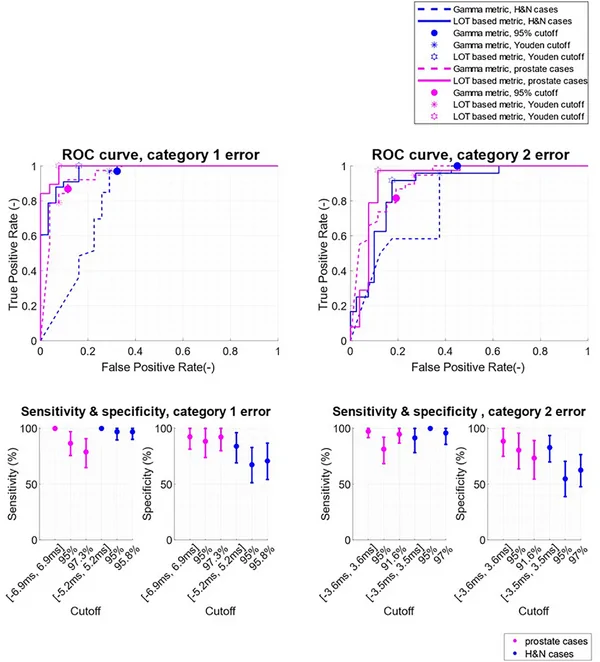
Receiver operating characteristic (ROC) curves for category 1 and 2 error detection. The Youden cutoff, the clinically used 95% gamma passing rate cutoff and their corresponding sensitivity and specificity are represented in the graphs.

**TABLE 3:** AUCs, *p*‐values related to Delong statistics for AUC comparison and Youden cutoffs.

Category	Location	Metric	AUC	Delong statistic *p*‐value at 5% significance level	Youden cutoff
1	Prostate	LOT‐based	0.99	0.16	[−6.9 ms, 6.9 ms]
		Gamma index	0.95		97.3%
	H&N	LOT‐based	0.97	$4*10^{-3}$	[−5.2 ms, 5.2 ms]
		Gamma index	0.82		95.8%
2	Prostate	LOT‐based	0.92	0.84	[−3.6 ms, 3.6 ms]
		Gamma index	0.91		91.6%
	H&N	LOT‐based	0.88	0.17	[−3.5 ms, 3.5 ms]
		Gamma index	0.80		97%

## 4 DISCUSSION

This work expands the LOT‐based metric, previously developed by Schopfer et al.^47^ by incorporating LOT‐based indices specific to OARs and analyzing the metric performance in detecting clinically relevant errors. For this, LOT discrepancies were introduced in H&N and prostate patient plans. The plans were delivered to an Octavius® II phantom and a check was subsequently performed to ensure proper deliveries. The LOT‐based metric indices were calculated and their ability to detect clinically relevant errors were analyzed and compared to the benchmark gamma index metric.

The results show good agreement between $LOT^{\mathrm{actual}}$ and $LOT^{\mathrm{ref}}$ as well as between $LOT^{\mathrm{actual}}$ and $LOT^{\mathrm{mod}}$ (Figure S1 in Supporting information), demonstrating consistency in plan deliveries. The mean LOT discrepancies of reference plans delivery, ranging between 1.6 ms and 1.9 ms, agree with previous results^45^ which showed mean LOT discrepancies in the range of [1.5 ms; 2.2 ms] between planned LOTs and LOTs measured with in phantom ionization chambers. These results also agree with Corradini et al.^53^ which observed a systematic shift of 2.0 ms between planned LOTs and LOTs measured with a Delivery Analysis (DA) software. The mean LOT discrepancies of modified plan deliveries show a wider range than for reference plans, with values between −1.6 ms and 3.6 ms. This is due to, for some modified plans, the introduction of artificial discrepancies in the LOT sinogram increasing the number of LOTs or Leaf Closed Times (LCTs, i.e. time between 2 consecutive opening of a leaf) falling below 60 ms, which is a regime where the MLC can generate larger LOT discrepancies. Therefore, to prevent complications in patient plan delivery, the TPS used in our clinic does not allow LOTs and LCTs below 60 ms. The standard deviations, ranging between 1.0 ms and 5.3 ms, align with Sevillano et al.,^40^ which observed values between 3.2 ms and 5.8 ms for different locations. Westerly et al.^38^ affirm that discrepancies between planned and delivered LOTs may arise from mechanical constraints on leaf motion and delays in the electronics that control the MLC. Han et al.^43^ reported that large LOT standard deviations occur for plans with many small LOTs. They also suggested that small MLC fields and steep movements may be a reason for large LOT standard deviations.

Schopfer et al.^47^ demonstrated that the mean LOT discrepancy is a reliable predictor of dose deviations in the PTV. This work strengthened the validity of the mean LOT discrepancy predictors by demonstrating their reliability in predicting dose deviations in the PTV, CTV, and OARs for H&N and prostate cases (Section 3.1). However, contrary to their results, our results show strong correlations between the gamma passing rate and the dose deviations in the PTV, as well as in the CTV and the OARs, positioning it as a reliable predictor. This disagreement may arise from the fact that, because the gamma passing rate values don't give information on the sign of the dose deviation, we focused on the relationship between the gamma passing rate values and the absolute dose deviation, in contrast to Schopfer et al.^47^ who focused on the sign. To the contrary, the LOT‐based metric indices provide information on the direction of the deviations; therefore, the sign was taken into account in this case. The gamma passing rate shows wider 95% confidence and prediction intervals than the LOT‐based metric indices. The exceptions to this are the brainstem and the spinal canal which have similar intervals, and the chiasm for which there is no linear correlation between the LOT‐based metric specific index and the dose deviation. This indicates that the gamma index metric exhibits greater variability and less precision in predicting dose deviations in most of the cases.

The results demonstrate that, in most of the cases, the LOT‐based metric indices provide stronger correlations with clinical goals compared to the gamma index metric (Section 3.2). However, the lack of significant correlation for certain LOT‐based metric indices shows that reliable predictors are missing for some OARs.

ROC curves analysis (Section 3.3) shows that both gamma index and LOT‐based metrics exhibit strong discrimination abilities in detecting category 1 errors. While their performances are similar for prostate cases, the LOT‐based metric outperforms the gamma index metric for H&N cases. Both metrics demonstrate good discrimination abilities in detecting category 2 errors, with comparable performances for both prostate and H&N cases. The clinically used 95% passing rate is not an optimal cutoff for category 1 and 2 error detection regarding Youden's index. Moreover, for prostate cases, the optimal cutoffs of the LOT‐based metric give better sensitivity than the optimal and the 95% cutoffs of the gamma index metric for both categories 1 and 2 error detection. For H&N cases, the optimal and 95% cutoffs of the gamma index metric show better sensitivity for category 2 error detection, while the optimal cutoffs of the LOT‐based metric show better sensitivity and specificity for category 1 and better specificity for category 2 error detection (Figure 4). To ensure safe deliveries, it is important to maximize specificity in order to correctly identify unacceptable plans and prevent them from being delivered to the patient. However, increasing specificity may also lead to decreasing the sensitivity, which implies unnecessary replanning of acceptable treatment plans. In a clinical setting, unnecessary replanning places additional burden to the workload. Therefore, the Youden's index used in this study helps achieve an optimal balance between specificity and sensitivity.

Limitations of this study include limited anatomical diversity and small sample size. This study focuses on H&N and prostate patients, excluding other anatomical localizations and thus limiting the generalizability of the findings. However, this focus enabled a targeted analysis of a localization that requires simple MLC modulation (prostate cases) and a localization that requires extensive MLC modulation, making it more susceptible to treatment delivery errors (H&N cases). Moreover, the selection criteria for H&N patients were intended to analyze a homogeneous cohort, while a wider spectrum of H&N cases would offer a more comprehensive analysis. This study includes a cohort of 8 patients, which is sufficient to identify preliminary trends, but increasing the cohort size would enhance the reliability of the findings. Furthermore, the calculation of LOT‐based metric indices represents a first‐order approximation, focusing exclusively on the beamlet delivering primary dose to the OAR and omitting the beamlets responsible solely of scattering events. Despite this limitation, the study demonstrates that focusing only on primary dose deposition beamlets already generates reliable predictors of clinically relevant delivery errors. Finally, the types of errors introduced in the LOT sinograms are limited; however, they were selected to represent typical MLC failures as well as worst‐case scenarios that could bypass the MLC interlock system.

In this study, we modified treatment plans by importing them into the “Create Machine QA” tab and manually modifying the sinogram. To deliver these modified plans, we generated machine QA procedures and executed the deliveries accordingly. As a result, the phantom was positioned arbitrarily on the treatment couch, rather than in the planned patient treatment position, and subsequently aligned with the green lasers. Consequently, the couch positions recorded in the raw data files during these deliveries do not reflect those that would occur in actual patient treatments. Because of this, we based our projection of the ROI volumes onto the MLC plane on the patient's planned treatment position rather than on the phantom delivery positions. Any errors occurring due to couch positioning were not taken into account in the LOT‐based metric indices calculation. However, for actual patient treatment deliveries, the couch positions recorded during delivery can be incorporated into the calculations. Errors in couch positioning would thus directly influence the LOT‐based metric indices. Looking ahead, particularly for treatments involving the Synchrony® system, it may be possible to collect the KVCT images acquired during delivery to determine the precise in‐treatment position of the ROIs. This approach could offer a more accurate alternative to relying solely on couch position data, as it would provide direct visual confirmation of ROI positioning and allow for more accurate projection onto the MLC plane. In contrast, this study takes into account jaw and gantry positions during delivery to determine the intersecting beamlets for the ROIs. Therefore, errors occurring in the jaw and gantry positions directly impact the LOT‐based metric indices.

This study validates a PSQA tool which offers a method to predict the accuracy of the delivery before treatment, thereby replacing the traditional clinical PSQA practice. It may also serve to analyze the TPS optimization robustness by prospectively evaluating the dosimetric impact of LOT discrepancies that may occur during delivery. Furthermore, this tool allows to retrospectively analyze the quality of delivered plans. This method is advantageous to using the LOT measurements directly, as rather than inputting them into a dose calculator, it eliminates the need for dose calculation software and accelerates the verification process. The PSQA based on LOT‐based metric provides an advantage over secondary dose calculations, which only evaluate dose accuracy without ensuring delivery validity and periodic dose measurements, which are not conducted for every treatment fraction.

The LOT‐based metric analysis is fully automated and does not require human intervention or material setup. It only requires access to a MATLAB® interpreter. It may be implemented in conventional and offline ART, by analyzing the delivery of the planned treatment plan in‐air prior to the treatment and by assessing the delivered treatment plan a posteriori. Furthermore, it may serve in online ART and motion tracking modalities by assessing the delivery of the adapted treatment plan a posteriori. Because the patient remains on the couch between the plan adaptation and the delivery in online ART workflow, the LOT‐based metric does not offer prospective plan approval. However, it can be used to track clinically significant errors occurring during delivery and account for them in subsequent adaptations, both in offline and online ART workflows. In online ART, it can also be used for robustness analysis of the adapted plan before delivery by assessing the risk of clinically relevant errors arising from typical LOT discrepancies caused by MLC mechanical constrains. Monitoring on a fraction‐by‐fraction basis enhances PSQA by offering the possibility of verifying both individual and overall treatment deliveries.

## 5 CONCLUSION

In this work, the LOT‐based metric was improved and its ability to detect clinically relevant delivery errors was evaluated compared against the benchmark gamma index metric. This study establishes the LOT‐based metric as a reliable tool for pretreatment PSQA, serving as a substitute for the conventional gamma index metric, while additionally offering the advantage of fraction‐by‐fraction monitoring of treatment delivery quality. In this study, the LOT‐based metric outperforms the gamma index metric in predicting dose deviations as well as clinical goal deviations. The LOT‐based metric also demonstrates superior performance in classifying acceptable and unacceptable plans in terms of dose deviation detection for H&N patients, while its performance in dose deviation detection for prostate cases and clinical goal deviation detection for both groups is comparable. The LOT‐based metric, with optimal cutoffs, outperforms the gamma index metric in terms of both sensitivity and specificity for dose deviation and clinical goal deviation detection in prostate cases, while for H&N cases, the gamma index metric outperforms the LOT‐based metric in term of sensitivity for clinical goal deviation detection.

## FUNDING INFORMATION

The authors have nothing to report.

## CONFLICT OF INTEREST STATEMENT

This research was funded by Accuray Inc. Edward Chao and Dylan Casey are employees of Accuray Inc.

## Supporting information



Supporting Information
