# Supplementary material for: Validation of a patient‐specific quality assurance tool for offline analysis of treatment deliveries
Source: Med Phys. 2025 Nov 25;52(12):e70161. doi: 10.1002/mp.70161 (PMC13733651; doi:10.1002/mp.70161)
Supplement: Supplementary file 1 [file MP-52-0-s001.doc]

# **Supplementary material**

# 1. Radixact® system

The Radixact® (Accuray Inc., Sunnyvale, USA) treatment unit makes use of a 6MV Flattening Filter Free (FFF) photon beam to deliver Intensity Modulation Radiation Therapy (IMRT) treatments. In brief, jaws are adjusted to collimate the field width in the cranio-caudal direction, with three available settings creating 1cm, 2.5cm, and 5cm field widths at the isocenter plane. A dynamic jaws mode can also modify the field width during treatment to minimize penumbra effects at the cranial and caudal target edges. The field size in the left-right direction is collimated by a binary MLC, composed of 64 leaves, with a maximum width of 40 cm at the isocenter plane. Each opened leaf produces an individual beamlet.

During delivery, MLC control relies on the LOT sinogram containing the planned LOTs. The LOT sinogram is a data array with one row per leaf and one column per projection. The actual LOTs may deviate from the planned ones due to mechanical constraints. Therefore, an interlock system stops irradiation if 20 or more MLC position-based errors occur. Position-based errors include a leaf failing to start a transition within 40ms or failing to reach the final position within 70ms of receiving an open/close command respectively^35^. However, this does not mitigate clinically relevant dose discrepancies, especially for small LOTs (below 50-60ms)^38^.

A megavoltage (MV) detector, composed of beam-focused ionization chambers, is mounted on the gantry ring opposite to the beam’s source^33^. This consists of 640 channels in which 576 record the exit fluence per linac pulse at a sampling rate of 300 Hz.

# 2. Reference and modified plan deliveries

# Figure S1 shows the discrepancies between and for two consecutive reference plan deliveries, as well as the discrepancies between and for the delivery of modified plans for patients P1 to P8.

# For the reference plan deliveries, the results show mean and median LOT discrepancies between 1.6ms and 1.9ms. The standard deviations stand between 1.0ms and 5.3ms. The unpaired two-sample t-tests show no significant differences between the mean LOT discrepancies of two consecutive deliveries of the same reference plan at 5% significance level (, ), except for patient P6, which shows a statistically significant difference (, ), with a mean LOT discrepancy of 1.88ms for the first delivery and of 1.83ms for the second delivery.

For the modified plan deliveries, the mean LOT discrepancies are between -1.6ms and 3.6ms, the medians between -1.3ms and 2.3ms and the standard deviations between 0.9ms and 6.2ms.

# Figure S1: Discrepancies between and for the delivery of reference plans and between and for the delivery of modified plans for all patients (P1 to P8).

# 3. Reference and modified plan deliveries

**Category 1 error, prostate cases**

Figure S2: First column: Relationship between evaluated dose parameters and gamma passing rates for the target and OARs of prostate cases. Second column: Relationship between dose parameters and LOT-based metric indices for the target and OARs of prostate cases. The red points represent the delivery of reference plans and the black points represent the delivery of modified plans. The plain red lines represent the dose discrepancy limits for category 1 error detection. The points in the dose range fulfill the dose limit for the evaluated dose parameter while the points outside this range fail to achieve the limit. The plain black lines correspond to the linear fit, for which only the data points with a dose discrepancy in the range of [-6%, +6%] were accounted for to ensure a good fit close to dose threshold. The dashed red lines correspond to the 95% gamma metric cutoff used in clinic.

**Category 1 error, H&N cases**

Figure S3: First column: Relationship between evaluated dose parameters and gamma passing rates for the target and OARs of H&N cases. Second column: Relationship between dose parameters and LOT-based metric indices for the target and OARs of H&N cases. The red points represent the delivery of reference plans and the black points represent the delivery of modified plans. The plain red lines represent the dose discrepancy limits for category 1 error detection. The points in the dose range fulfill the dose limit for the evaluated dose parameter while the points outside this range fail to achieve the limit. The plain black lines correspond to the linear fit, for which only the data points with a dose discrepancy in the range of [-6%, +6%] were accounted for to ensure a good fit close to dose threshold. The dashed red lines correspond to the 95% gamma metric cutoff used in clinic.

**Category 2 error, prostate cases**

# Figure S4: First column: Relationship between the gamma passing rate and the evaluated clinical goals for target and OARs of prostate cases. Second column: Relationship between the LOT-based metric indices and the evaluated clinical goals for target and OARs of prostate cases. The red points represent the delivery of reference plans and the black points represent the delivery of modified plans. The evaluated clinical goals are listed in table 2. The dose constraints have been divided by the total number of fractions to display the constraint for a unique delivery. The red dashed lines represent the 95% gamma metric cutoff used in clinic. The red plain lines represent the acceptance level.

**Category 2 error, H&N cases**


Figure S5: First column: Relationship between the gamma passing rate and the evaluated clinical goals for target and OARs of H&N cases. Second column: Relationship between the LOT-based metric indices and the evaluated clinical goals for target and OARs of H&N cases. The red points represent the delivery of reference plans and the black points represent the delivery of modified plans. The evaluated clinical goals are listed in table 2. The dose constraints have been divided by the total number of fractions to display the constraint for a unique delivery. The red dashed lines represent the 95% gamma metric cutoff used in clinic. The red plain lines represent the acceptance level.

# 4. Sensitivity and specificity of gamma index metric and LOT-based metric

| **Error** | **Localization** | **LOT-based metric** | | | **Gamma index metric** | | |
| --- | --- | --- | --- | --- | --- | --- | --- |
|  |  | **Cutoff**  **(ms)** | **Sensitivity (%)** | **Specificity**  **(%)** | **Cutoff**  **(%)** | **Sensitivity**  **(%)** | **Specificity**  **(%)** |
| **Category 1** | Prostate | [-6.9;6.9] | 100  CI [100;100] | 92.5  CI [81.4;100] | 95 | 86.6  CI [75.7;97.1] | 88.4  CI [73.9;100] |
|  |  |  |  |  | 97.3 | 78.9  CI [64.8;90.8] | 92.3  CI [80;100] |
|  | H&N | [-5.2;5.2] | 100  [100;100] | 84.0  [69.1;96.2] | 95 | 97.0  CI [89.7;100] | 67.4  CI [51.3;82.9] |
|  |  |  |  |  | 95.8 | 96.9  CI [90.2;100] | 70.8  CI [54.2;86.8] |
| **Category 2** | Prostate | [-3.6;3.6] | 97.4  CI [91.7;100] | 88.5  CI [75;100] | 95 | 81.4  CI [68.4;92.1] | 80.5  CI [63.8;95.7] |
|  |  |  |  |  | 91.6 | 94.8  CI [86.8;100] | 73.4  CI [54.5;89.1] |
|  | H&N | [-3.5;3.5] | 91.5  CI [78.3;100] | 82.9  CI [69.9;93.6] | 95 | 100  CI [100;100] | 54.8  CI [38.8;70.5] |
|  |  |  |  |  | 97 | 95.9  CI [85.7;100] | 62.6  CI [47.7;76.5] |

Table S1: Sensitivity, specificity, and corresponding CI for different cutoffs and calculated with the Bootstrap method. Those values are represented in the second line of figure 4.
